# Effects of visual art therapy on depressive symptoms in adults: a systematic review and meta-analysis

**DOI:** 10.3389/fpsyt.2026.1877502

**Published:** 2026-07-22

**Authors:** Zhenhua Guo, Yingcong Liu, Shan Zhu, Yupeng He

**Affiliations:** 1 Department of Design, Dong-A University, Busan, Republic of Korea; 2 School of Music Education, Wuhan Conservatory of Music, Wuhan, China; 3 School of Pharmaceutical Sciences, Wenzhou Medical University, Wenzhou, China

**Keywords:** adults, anxiety symptoms, depressive symptoms, meta-analysis, systematic review, visual art therapy

## Abstract

**Objective:**

To systematically evaluate the intervention effects of visual art therapy on depressive symptoms in adults, and to examine its impact on anxiety symptoms.

**Methods:**

A systematic search was conducted in databases including CNKI, WanFang Data, VIP, Chinese Biomedical Literature Database (CBM), PubMed, Web of Science, Cochrane Library, and Embase from inception to March 2026. Randomized controlled trials were included in which visual art therapy was used to intervene in depressive symptoms among adults (≥18 years).Meta-analysis was performed using Stata 18.0 software.The primary outcome was depressive symptoms, measured by scales such as the BDI, GDS, HADS-D, and SDS, and the secondary outcome was anxiety symptoms, measured by scales such as the BAI, HADS-A, and SAS. Fixed-effect or random-effect models were selected according to the level of heterogeneity, and subgroup analyses were conducted.

**Results:**

A total of 12 randomized controlled trials involving 741 adults were included, with 377 participants in the intervention group and 364 in the control group.The meta-analysis showed that visual art therapy effectively alleviated depressive symptoms (SMD = -0.81, 95% CI: -1.16 to -0.46, P < 0.00001) and anxiety symptoms (SMD = -0.69, 95% CI: -0.90 to -0.48, P < 0.00001) in adults.Subgroup analyses indicated that interventions with a duration of <12 weeks, <12 sessions in total, and a single-session length of ≤60 minutes were associated with larger effect sizes for the improvement of depressive symptoms; improvements in anxiety symptoms were more pronounced in intervention protocols characterized by higher frequency (>12 sessions), shorter overall duration (≤6 weeks), and shorter single-session length (≤60 minutes).

**Conclusion:**

As a non-pharmacological intervention, VAT has potential as an adjunctive approach for alleviating depressive and anxiety symptoms in adults.

**Systematic review registration:**

https://www.crd.york.ac.uk/PROSPERO/view/CRD420261371354, identifier CRD420261371354.

## 1 Introduction

Depression is one of the leading mental disorders contributing to disability-adjusted life years (DALYs) lost worldwide (1). According to the World Health Organization, the global number of individuals with depression exceeded 320 million in 2024, accounting for 4.0% of the world’s population, with the proportion reaching 5.7% among adults (2). Depressive symptoms are the core manifestation of depressive disorders (3) and are also highly prevalent in subclinical populations and among patients with chronic physical illnesses (4, 5). In adulthood, depressive symptoms not only severely impair emotional regulation, social functioning, and occupational capacity, but also show high comorbidity with cardiovascular disease, diabetes, and suicidal behavior, thereby imposing a substantial public health burden (6, 7). In addition, the comorbidity rate of depressive and anxiety symptoms is as high as 45.7%–70.0% (8), and the two mutually exacerbate each other, leading to poorer treatment outcomes and a higher risk of relapse (9).

At present, pharmacological treatments for alleviating depressive symptoms mainly include selective serotonin reuptake inhibitors (SSRIs) and related agents (2). However, pharmacotherapy is associated with several limitations, including delayed onset of action (typically 4–6 weeks), significant side effects (e.g., weight gain, sexual dysfunction), and poor adherence to long-term medication (10, 11). When depressive and anxiety symptoms coexist, both the response rate and clinical remission rate to standard antidepressant treatment are significantly reduced (12). Existing evidence indicates that combined pharmacotherapy and psychotherapy is significantly more effective than pharmacotherapy alone (13). Against this background, psychological interventions such as behavioural activation, cognitive behavioural therapy, interpersonal psychotherapy, and music therapy (2, 14) have been widely recognized as effective adjunctive or alternative options for the treatment of depression. In recent years, visual art therapy (VAT), as an emerging non-pharmacological psychological intervention, has shown promising potential for application in improving depressive symptoms due to its unique modes of expression and therapeutic mechanisms (15).

VAT is a form of psychological intervention that uses visual art media such as drawing, coloring, collage, and sculpture as core tools. Under the professional guidance of a therapist, art-making is deliberately employed to mobilize imagery, color, form, and material qualities as a nonverbal channel for emotional expression and regulation, thereby facilitating emotional expression, self-exploration, and meaning-making (16). Randomized controlled trials have shown that VAT can significantly improve depressive symptoms in older women with depression (17). Specifically, Zentangle-based interventions significantly alleviated depressive symptoms in community-dwelling older adults with depression, with the therapeutic effects persisting up to six weeks after the intervention (18); moreover, mandala group art therapy significantly reduced depressive scores among bereaved college students (19). In addition, VAT has also been found to alleviate anxiety symptoms in adults with major depressive disorder (20).

To date, only one systematic review and meta-analysis (21) has synthesized studies published up to 2023 and provided evidence for the efficacy of VAT in alleviating depressive symptoms in adults. However, meta-analytic evidence remains insufficient with respect to eligibility criteria, treatment parameters, intervention modalities, and study populations. Specifically, the following issues remain: (1) randomized controlled trials and adult samples aged ≥18 years have not been strictly required; (2) key “dose parameters” such as total number of sessions and single-session duration have not been adequately captured; (3) Considerable variability in intervention modalities leads to uncertainty regarding the comparative efficacy of different VAT subtypes; (4) potential differences in treatment effects across populations (e.g., individuals with depressive disorders, post-stroke populations, and community samples) have yet to be fully explored; (5) anxiety, a major comorbid outcome of depressive symptoms, has not been incorporated into the analyses, limiting a comprehensive evaluation of the clinical effectiveness of VAT; and (6) recently published studies in the past three years have not been included, thereby weakening the timeliness and completeness of the existing synthesized evidence. Therefore, this study conducted a comprehensive search of Chinese and English databases and included randomized controlled trials, with depression as the primary outcome and anxiety as the secondary outcome, to quantitatively synthesize the effects of VAT on depressive symptoms in adults. In addition, subgroup analyses were performed to examine the potential influences of intervention modality, treatment parameters, and population characteristics, with the aim of providing evidence-based recommendations for clinical decision-making.

## 2 Materials and methods

### 2.1 Registration

This systematic review and meta-analysis was prospectively registered in the International Prospective Register of Systematic Reviews (PROSPERO; registration number CRD420261371354) and was conducted in accordance with the Preferred Reporting Items for Systematic Reviews and Meta-Analyses (PRISMA) guidelines.

### 2.2 Search strategy

A systematic search was conducted in CNKI, Wanfang, VIP, the Chinese Biomedical Literature Database, PubMed, Web of Science, the Cochrane Library, and Embase, from database inception to March 2026. The search strategy was developed based on the research topic using a combination of subject headings and free-text terms. English search terms mainly focused on visual art therapy, adults, depression, and randomized controlled trials, and included “Art Therapy”, “Visual Art Therapy”, “painting”, “collage”, “sculpture”, “craft”, “adult”, “depression”, “depressive symptoms”, “depressive disorder”, “randomized controlled trial”, “RCT”, and other related terms.;The main Chinese search terms included “视觉艺术治疗” (visual art therapy), “艺术治疗” (art therapy), “绘画治疗” (painting therapy), “美术治疗” (fine art therapy), “拼贴” (collage), “雕塑” (sculpture), “陶艺” (ceramics), “成人” (adult), “抑郁” (depression), “抑郁症状” (depressive symptoms), “抑郁障碍” (depressive disorder),and other related terms. The search strategies were adapted to the specific search syntax of each database, and the detailed search strategies are provided in the **Supplementary Materials**.

### 2.3 Inclusion and exclusion criteria

Inclusion criteria: (1) Participants: adults aged ≥18 years who were reported in the original studies as having depressive symptoms, with no restrictions on sex, underlying physical conditions, medication status, or whether they met the formal diagnostic criteria for depressive disorders; (2) Intervention: the experimental group received interventions centered on visual art creation, including forms of visual art therapy such as drawing, collage, sculpture, ceramics, handicrafts, and coloring, delivered in either individual or group formats; (3) Control condition: the control group received usual care (e.g., maintenance of routine medication, general psychological support, daily recreational activities, physical therapy and rehabilitation training, and health education); (4) Study design: randomized controlled trials (RCTs); (5) Outcomes: at least one depression-related outcome was reported; (6) Language: studies published in Chinese or English were included.

Exclusion criteria: (1) studies with duplicate publications or overlapping data; (2) non-randomized controlled studies, such as narrative reviews, systematic reviews, case reports, conference abstracts, study protocols, and theoretical articles; (3) studies in which the intervention did not fall within the scope of visual art therapy, or in which visual art therapy was only part of a combined intervention and its independent effects could not be extracted; (4) studies in which participants were younger than 18 years; (5) studies for which the full text was unavailable or extractable data were lacking.

Outcomes: The primary outcome was depression, assessed using the Beck Depression Inventory-II (BDI-II), Beck Depression Inventory (BDI), Geriatric Depression Scale-15 (GDS-15), Geriatric Depression Scale-Short Form (GDS-SF), Hospital Anxiety and Depression Scale–Depression (HADS-D), Self-Rating Depression Scale (SDS), and other related scales. The secondary outcome was anxiety, assessed using the Beck Anxiety Inventory (BAI), Hospital Anxiety and Depression Scale–Anxiety (HADS-A), Self-Rating Anxiety Scale (SAS), and other related scales.

### 2.4 Data extraction

The retrieved records were imported into EndNote 21 for management and de-duplication. Based on the predefined inclusion and exclusion criteria, two researchers independently screened the literature, first by reading the titles and abstracts and then by reviewing the full texts to determine the final included studies; any disagreements were resolved through consultation with a third researcher. Data extraction was independently performed by two researchers using a standardized form, mainly including first author, year of publication, participant characteristics, sample size, intervention measures, control measures, intervention duration, length of each session, number of sessions and outcome measures. For outcome data, means, standard deviations, and sample sizes were primarily extracted; if the data were incomplete, they were collated using information from the original text, and studies from which data could still not be extracted were excluded from the quantitative synthesis. All data extraction procedures were completed prior to the conduct of the systematic review to ensure the accuracy and completeness of the data.

### 2.5 Risk of bias assessment

The Cochrane Risk of Bias tool (Version 1.0, ROB 1) was used to assess the methodological quality of the included randomized controlled trials. The assessment domains included random sequence generation, allocation concealment, blinding of participants and personnel, blinding of outcome assessment, incomplete outcome data, selective reporting, and other sources of bias. Each item was rated as “low risk”, “high risk”, or “unclear risk” of bias. A graded assessment approach was then applied to evaluate the overall credibility of the body of evidence.

### 2.6 Data synthesis and statistical analysis

This study utilised RevMan 5.4 and Stata 15.0 software to conduct a meta-analysis. For continuous data, the standardized mean difference (SMD) and its 95% confidence interval (CI) were used as effect sizes, and pooled analyses were conducted using the inverse-variance method. A P value < 0.05 was considered statistically significant. Between-study heterogeneity was assessed using the χ² test and the I² statistic; when I² > 50% or the P value of the χ² test was < 0.10, substantial heterogeneity was considered present and a random-effects model was applied, otherwise a fixed-effect model was used.

For outcome analyses, depression was treated as the primary outcome and anxiety as the secondary outcome, and separate meta-analyses were conducted for each. If a single study reported multiple scale-based results for depressive outcomes, data from the scale with more complete reporting and wider clinical application were preferentially extracted for inclusion in the analysis; if a single study reported both depressive and anxiety outcomes, data for each outcome were extracted and included in the corresponding analyses. Sensitivity analyses were conducted by sequentially excluding individual studies to examine the robustness of the pooled results. When at least 10 studies were included for a given outcome, funnel plots were generated and Egger’s regression test was used to assess publication bias.

## 3 Results

### 3.1 Search results

**Figure 1:**
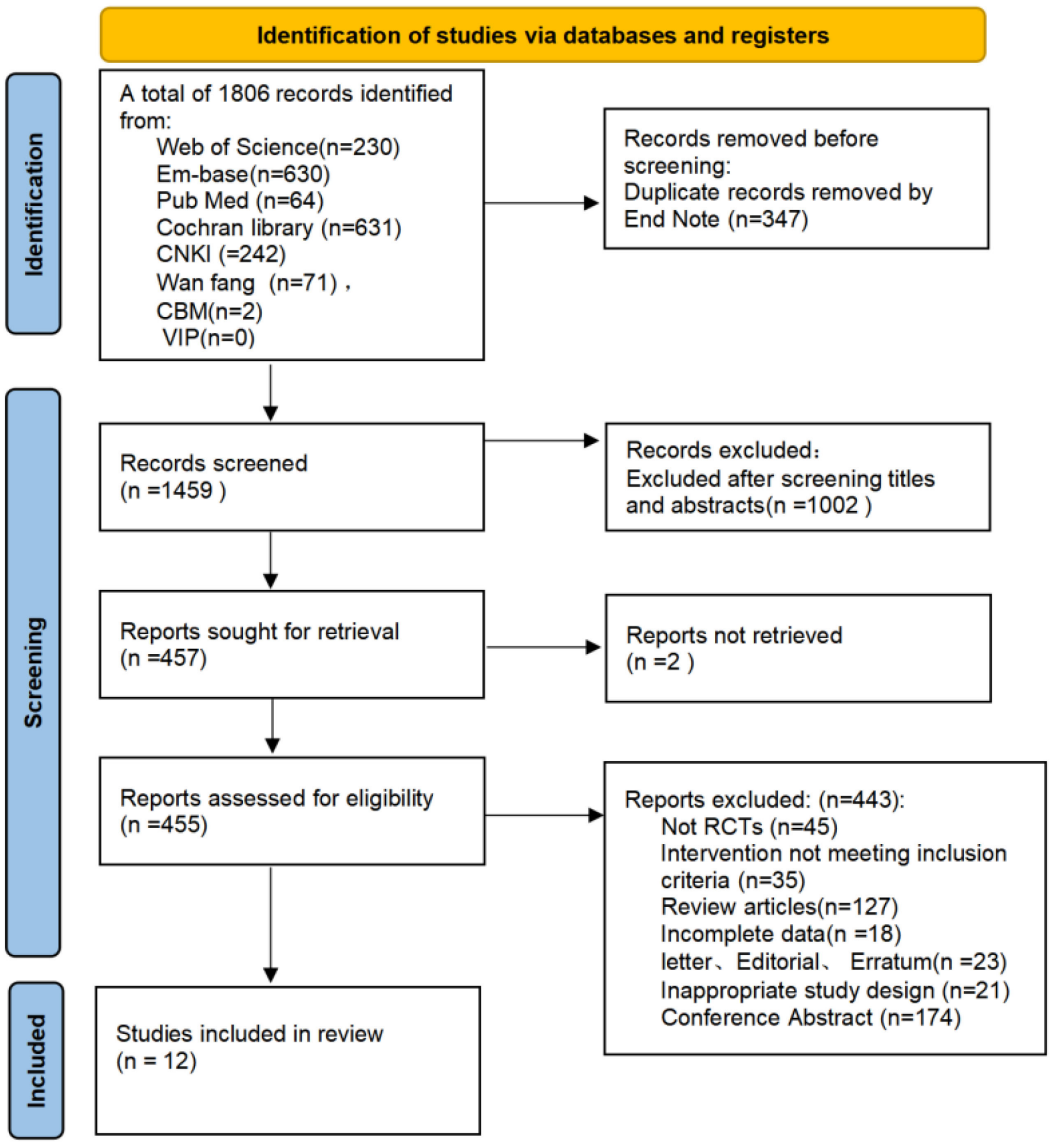
Flow diagram of study selection.

After systematic searching and stepwise screening, 12 studies in Chinese and English were finally included, comprising a total of 741 participants, with 377 in the intervention group and 364 in the control group. The study selection process is presented in **Figure 1**, and the basic characteristics of the included studies are summarized in **Table 1**.

**Table 1:** Characteristics of the included studies.

Source	Group size/age		Intervention description		Population	Treatment course (weeks)	Session duration (min)	Total number of treatments (session)	Outcomes
	IG	CG	IG	CG					
Bosomtwe Samuel 2022 (22)	43 46.56 ± 13.02	45 49.91 ± 11.11	Structured Pattern Drawing Therapy	Treatment as Usual	GAD	3	30-120	15	SDS, SAS
Caroline Ellis-Hill 2019 (23)	25 72.0 ± 11.22	22 67.4 ± 12.83	Multi-material Visual Art Therapy	usual care	Stroke Survivors	14	120	10	HADS-D, HADS-A
Ehteram Sadat Ilali 2019 (24)	27 70	27 70	Free Painting Therapy	Routine Daily Activities	Community-Based adults	6	60	6	GDS-15
Eliana C. Ciasca 2018 (17)	31 66.1 ± 5.7	25 69.8 ± 6.4	Multi-material Visual Art Therapy	Pharmacotherapy	Depression	20	90	20	GDS-15, BAI
Golden M. Masika 2022 (25)	51 73.1 ± 7.6	56 74.1 ± 8.3	Structured Pattern Drawing Therapy	Health Education	MCI	6	120	12	GDS-SF
Han Choi 2020 (20)	19 32.4	16 36.6	Free Painting Therapy	Pharmacotherapy	Depression	6	60	6	BDI-II, BAI
Havva Gezgin Yazici 2024 (26)	30 66.80 ± 9.96	30 61.20 ± 13.81	Clay Art Therapy	Routine Physical Therapy	Stroke Survivors	8	60	16	BDI
Joshua KM Nan 2017 (27)	52 46.1 ± 10.5	48 44.1 ± 10.0	Clay Art Therapy	Non-Directive Art Activities	Depression	6	150	6	BDI-II
Pei-Ching Tsai 2025 (28)	11 /	11 /	Multi-material Visual Art Therapy	Routine Community Services	Community-Based Adults	12	90	12	GDS-15
Se-Ryun Park 2023 (29)	18 52.4 ± 15.4	14 44.9 ± 14.4	Multi-material Visual Art Therapy	No-Intervention	adults who had lost an immediate family member within one year	8	60	8	BDI-II
Yang Liu 2025 (30)	30 35.73 ± 6.51	30 37.67 ± 7.37	Free Painting Therapy	Conventional Psychological Correction	Community-Based Adults	2	60	5	SDS, SAS
Yan Xu 2022 (31)	40 42.30 ± 15.76	40 41.15 ± 16.94	Structured Pattern Drawing Therapy	Treatment as Usual	Depression	4	60	8	SDS, SAS

IG, intervention group; CG, control group; GAD, Generalized Anxiety Disorder; MCI, mild cognitive impairment; SDS, Self-Rating Depression Scale; SAS, Self-Rating Anxiety Scale; HADS-D, Hospital Anxiety and Depression Scale - Depression subscale; HADS-A, Hospital Anxiety and Depression Scale - Anxiety subscale; GDS-15, Geriatric Depression Scale-15; BAI, Beck Anxiety Inventory; GDS-SF, Geriatric Depression Scale - Short Form; BDI-II, Beck Depression Inventory-II; BDI, Beck Depression Inventory.

Structured Pattern Drawing Therapy: characterised by fixed templates, repetitive patterns, limited freedom, and an emphasis on relaxations; Free Painting Intervention Therapy: uses painting as the core medium, with no fixed templates and relatively free choice of subject matter; Multi-material Visual Art Therapy: involves the use of two or more art media, or includes non-painting materials (such as collage, photography and weaving.

### 3.2 Risk of bias assessment

**Figure 2:**
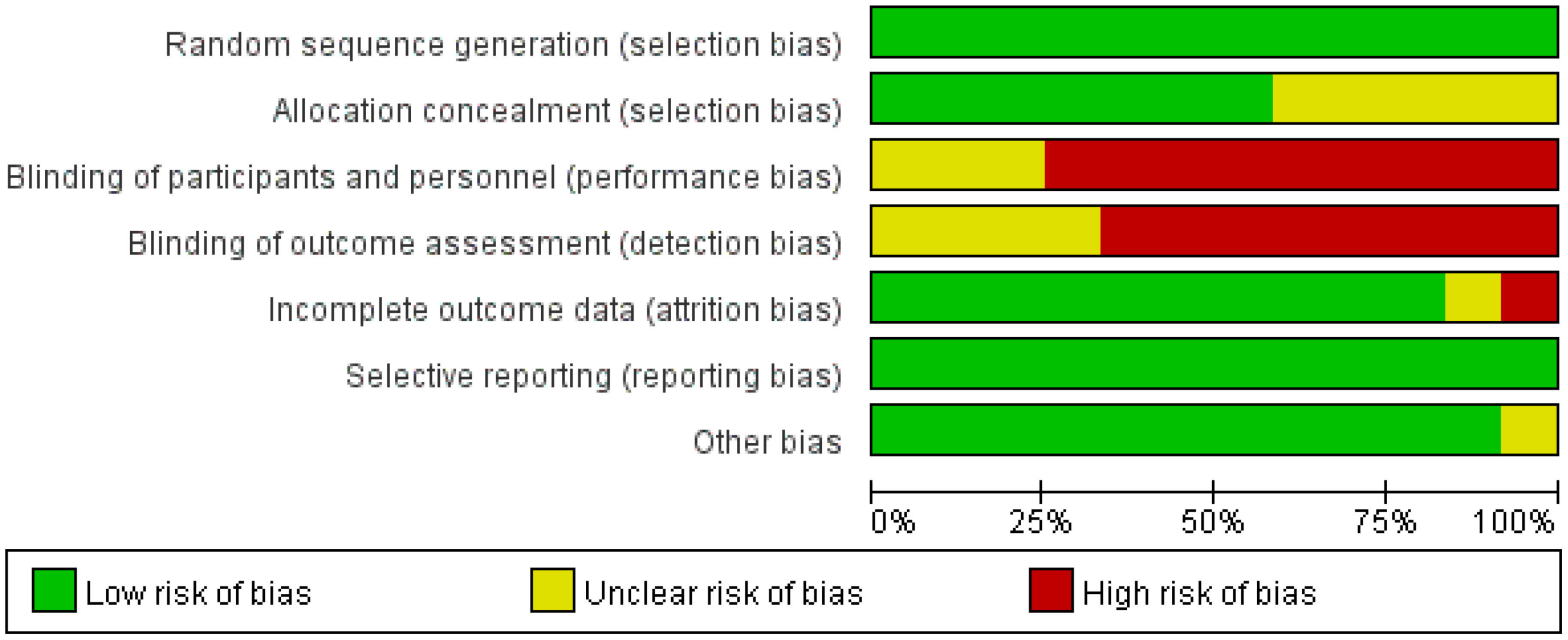
Risk of bias assessment of the included studies.

**Figure 3:**
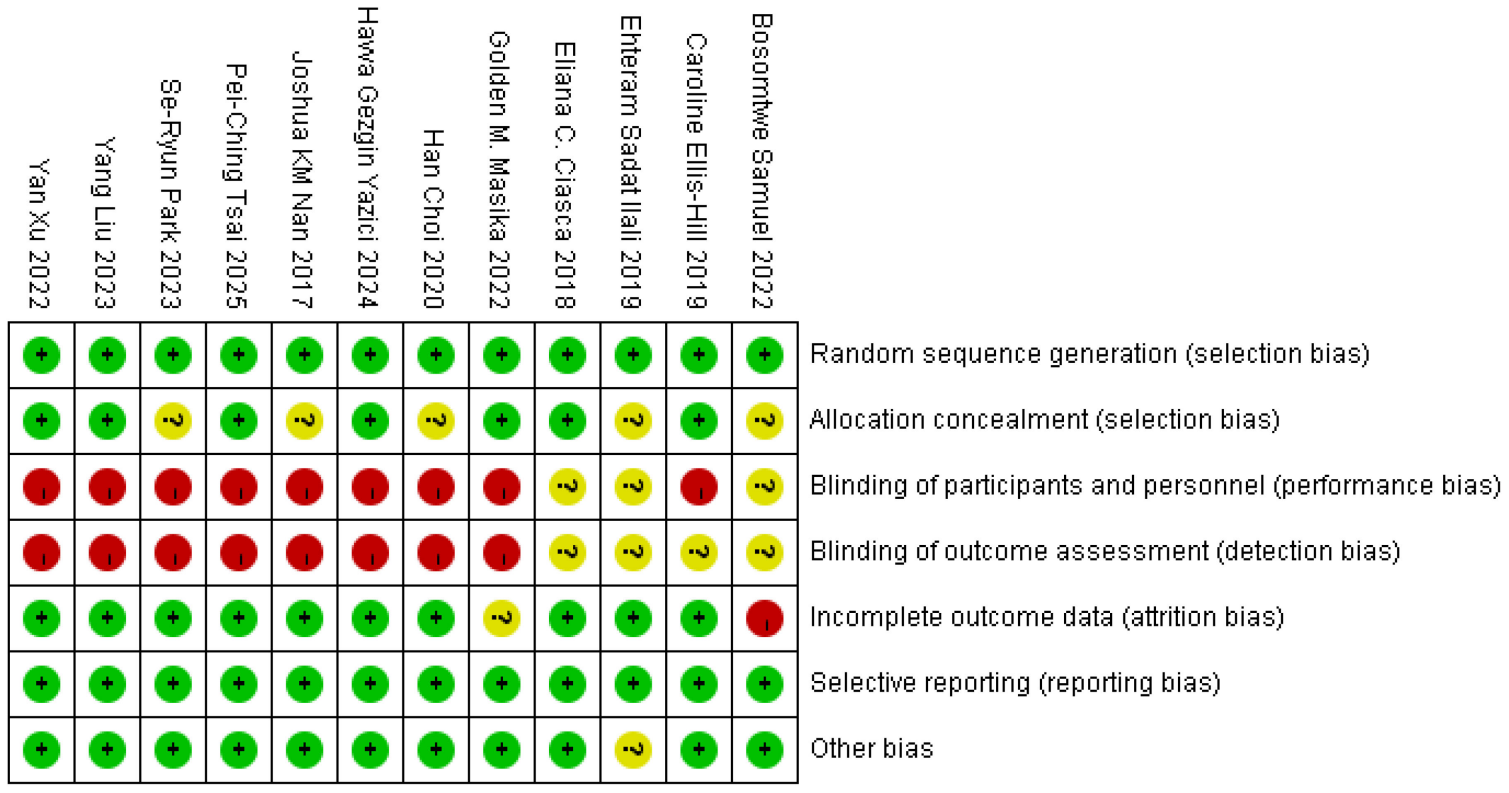
Risk of bias summary for the included studies.

The Cochrane Risk of Bias tool was used to assess the methodological quality of the 12 included randomized controlled trials, and the results are shown in **Figures 2**, **3**. A total of seven studies explicitly reported appropriate methods of randomization, including the lottery method, random number tables, or sealed envelopes. Only one study reported participant attrition. No obvious additional sources of bias were identified in any of the studies. Overall, the main sources of bias in the included studies were related to blinding, while the risk of bias for the other domains was generally low. Using a graded assessment approach, the overall credibility of the evidence was rated as “moderate” for depressive outcomes and “low” for anxiety outcomes. Detailed ratings of the certainty of evidence are provided in **Supplementary Material S2**.

### 3.3 Meta-analysis results

#### 3.3.1 Effects of visual art therapy on depressive symptoms in adults

**Figure 4:**
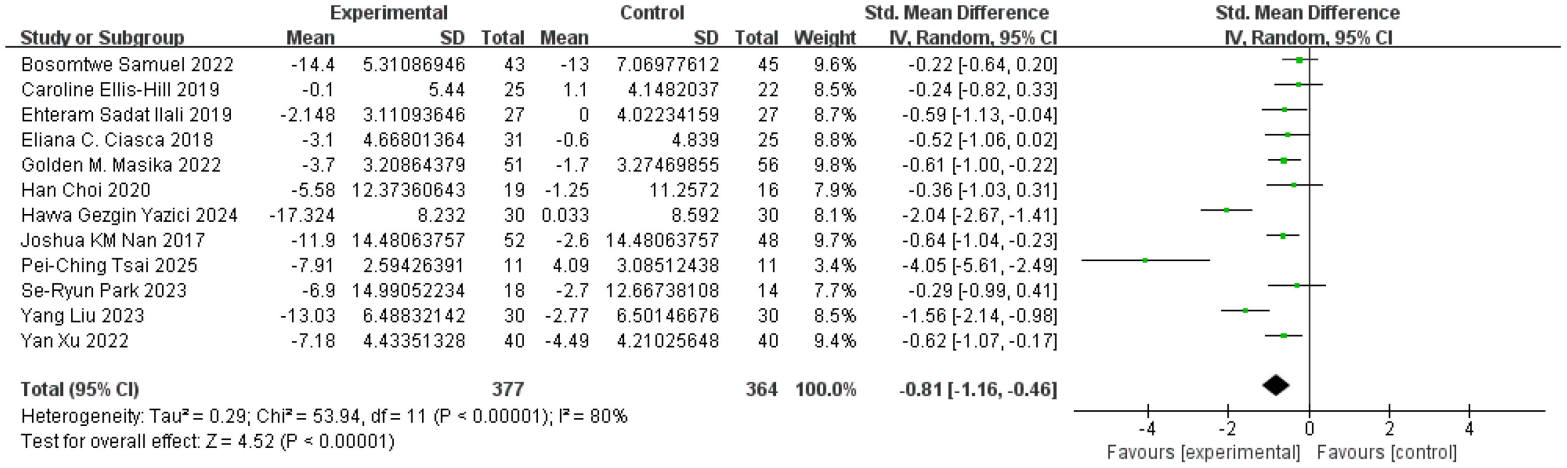
Forest plot of the effects of visual art therapy on depressive symptoms in adults.

**Figure 5:**
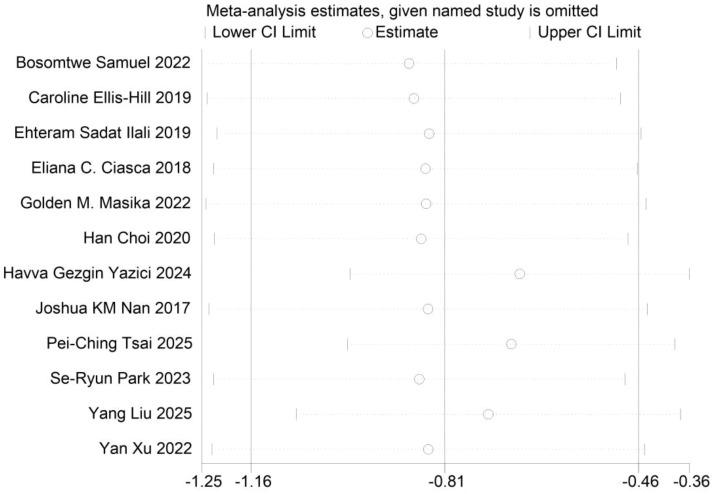
Sensitivity analysis of the effects of visual art therapy on depressive symptoms in adults.

**Figure 6:**
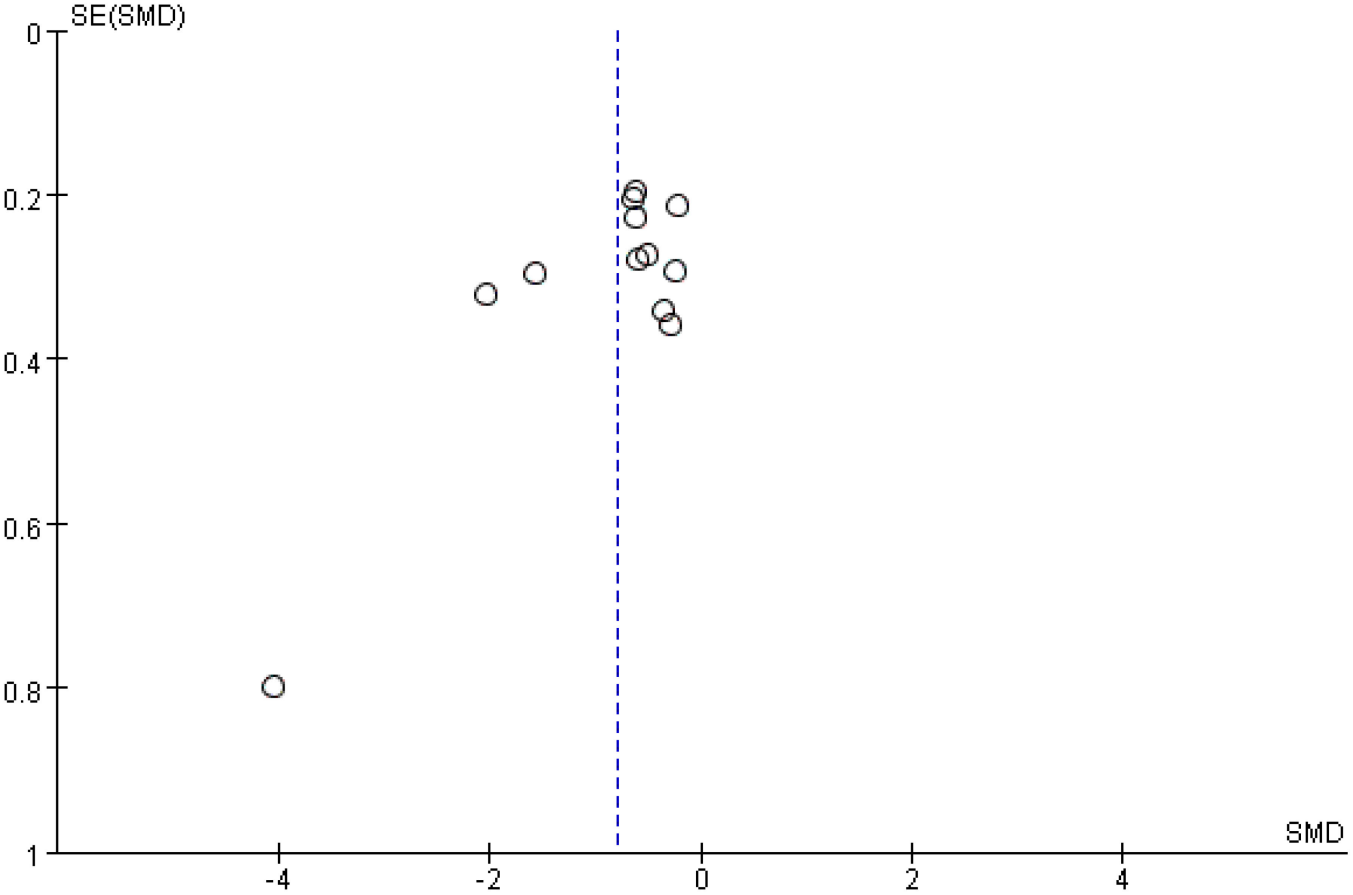
Funnel plot of the effects of visual art therapy on depressive symptoms in adults.

The meta-analysis of 12 studies (**Figure 4**) showed that the depression scores in the visual art therapy group were lower than those in the control group, and the difference was statistically significant (SMD = -0.81, 95% CI: -1.16~-0.46, Z = 4.52, P < 0.00001). The heterogeneity test indicated substantial heterogeneity among studies (Tau² = 0.29, χ² = 53.94, df = 11, P < 0.00001, I² = 80%); therefore, a random-effects model was used for pooling. Sensitivity analyses (**Figure 5**) showed that sequential exclusion of individual studies did not materially change the direction or statistical significance of the pooled effect size, suggesting that the results were robust. The funnel plot (**Figure 6**) showed that the scatter of the included studies was basically symmetrical. The Egger’s test was further conducted, and the result showed no statistically significant difference (P = 0.054), indicating that no significant publication bias was detected. However, due to the limited number of included studies, the statistical power of this test may be insufficient to rule out the possibility of publication bias.

#### 3.3.2 Effects of visual art therapy on anxiety symptoms in adults

**Figure 7:**
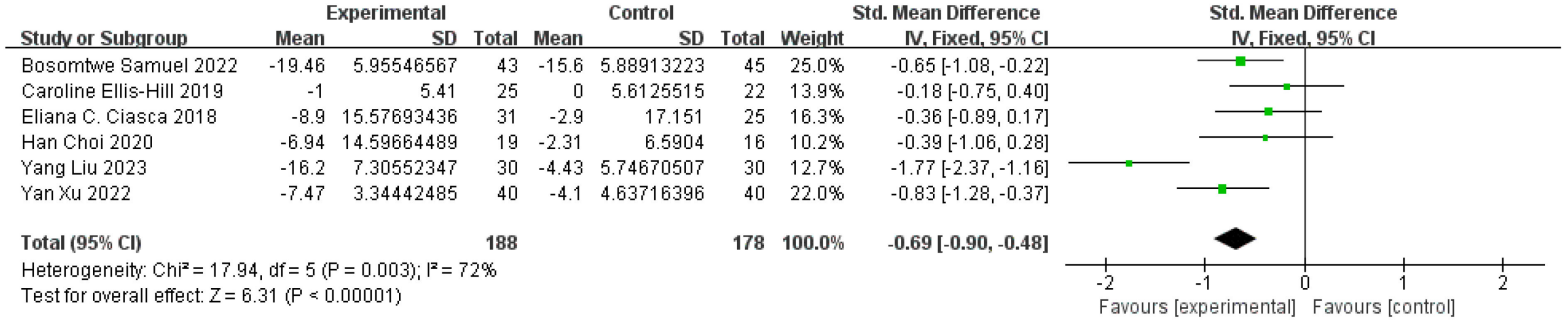
Forest plot of the effects of visual art therapy on anxiety symptoms in adults.

A total of six studies involving 366 participants were included, with 188 in the intervention group and 178 in the control group. A meta-analysis was conducted with anxiety as the outcome, and the results (**Figure 7**) showed that anxiety scores in the visual art therapy group were significantly lower than those in the control group (SMD = -0.69, 95% CI: -0.90~-0.48, Z = 6.31, P < 0.00001). The heterogeneity test indicated substantial heterogeneity among the studies (χ² = 17.94, df = 5, P = 0.003, I² = 72%), suggesting a certain degree of inconsistency in the study results.

### 3.4 Subgroup analyses

#### 3.4.1 Subgroup analyses of the effects of visual art therapy on depressive symptoms in adults

To explore potential sources of heterogeneity, subgroup analyses were conducted according to treatment duration (weeks), number of sessions, length of each session, type of intervention, target population, and age (see **Table 2**). In terms of treatment duration, both ≤6 weeks (SMD = -0.64, 95% CI: -0.92 to -0.36, P < 0.0001) and 7–12 weeks (SMD = -2.00, 95% CI: -3.73 to -0.27, P = 0.02) showed significant improvements, whereas >12 weeks (SMD = -0.39, 95% CI: -0.78 to 0.00, P = 0.05) was at a borderline level.

**Table 2:** Subgroup analyses of depressive outcomes associated with visual art therapy in adults.

Subgroups	No. of studies	Total sample (I+C)	Heterogeneity		SMD (95% CI)	Meta-analysis results	
			I^2^ (%)	P-value		Z	P
Treatment duration (weeks)							
≤6	7	524	57	0.03	-0.64 [-0.92, -0.36]	4.52	< 0.0001
7-12	3	114	92	< 0.0001	-2.00 [-3.73, -0.27]	2.26	0.02
>12	2	103	0	0.49	-0.39 [-0.78, 0.00]	1.95	0.05
Treatment Frequency (sessions)							
<8	4	249	68	0.02	-0.79 [-1.26, -0.31]	3.23	0.001
8-12	5	288	81	0.00003	-0.81 [-1.42, -0.19]	2.57	0.01
>12	3	204	91	< 0.0001	-0.90 [-1.92, 0.11]	1.74	0.08
Session Duration (min)							
≤60	5	267	83	< 0.0001	-0.98 [-1.63, -0.32]	2.93	0.003
>60	5	323	80	0.0004	-0.82 [-1.37, -0.26]	2.88	0.004
Type of Intervention							
CAT	2	160	93	0.0002	-1.31 [-2.68, 0.06]	1.88	0.06
SPDT	3	275	12	0.32	-0.48 [-0.73, -0.2]	3.95	< 0.0001
FPT	3	149	77	0.01	-0.84 [-1.56, -0.1]	2.3	0.02
MVAT	4	157	86	0.0001	-0.98 [-1.94, -0.02]	2.01	0.04
Population							
D	4	271	0	0.90	-0.57 [-0.81, -0.33]	4.58	< 0.0001
SS	2	107	94	< 0.0001	-1.13 [-2.89, 0.62]	1.26	0.21
CBA	3	136	89	< 0.0001	-1.84 [-3.19, -0.49]	2.68	0.007
Ages							
<65	7	455	82	< 0.0001	-0.81 [-1.27, -0.34]	3.37	0.0007
≥65	5	286	89	0.0004	-0.83 [-1.43, -0.23]	2.73	0.006

I+C, intervention group plus control group; SMD, standardized mean difference; CI, confidence interval; CAT, clay art therapy; SPDT, structured pattern drawing therapy; FPT, free painting therapy; MVAT, multi-media visual art therapy; D, adults with depression; SS, post-stroke survivors; CBA, community-based adults.

Regarding the number of sessions, both <8 sessions (SMD = -0.79, 95% CI: -1.26 to -0.31, P = 0.001) and 8–12 sessions (SMD = -0.81, 95% CI: -1.42 to -0.19, P = 0.01) were statistically significant, whereas >12 sessions did not reach statistical significance (SMD = -0.90, 95% CI: -1.92 to 0.11, P = 0.08). With respect to the length of each session, both ≤60 minutes (SMD = -0.98, 95% CI: -1.63 to -0.32, P = 0.003) and >60 minutes (SMD = -0.82, 95% CI: -1.37 to -0.26, P = 0.004) were statistically significant.

When stratified by type of intervention, structured pattern drawing therapy (SMD = -0.48, 95% CI: -0.73~-0.20, P < 0.0001), free drawing therapy (SMD = -0.84, 95% CI: -1.56~ -0.10, P = 0.02), and multimodal visual art therapy (SMD = -0.98, 95% CI: -1.94~-0.02, P = 0.04) all showed statistically significant effects. Clay art therapy (SMD = -1.31, 95% CI: -2.68 to 0.06, P = 0.06) did not reach statistical significance.

When stratified by target population, significant improvements were observed both in individuals with depression (SMD = -0.57, 95% CI: -0.81~-0.33, P < 0.0001) and in community-dwelling adults (SMD = -1.84, 95% CI: -3.19~-0.49, P = 0.007).In contrast, the subgroup of post-stroke survivors did not show a statistically significant difference(SMD = -1.13, 95% CI -2.89~0.62, P = 0.21). Age-stratified analyses showed statistically significant effects in both participants aged <65 years(SMD = -0.81, 95% CI -1.27, -0.34, P = 0.0007)and those aged ≥65 years(SMD = -0.83, 95% CI -1.43,-0.23, P = 0.006), with similar effect sizes between the two groups.

#### 3.4.2 Subgroup analyses of the effects of visual art therapy on anxiety outcomes in adults

To explore potential sources of heterogeneity, subgroup analyses were conducted according to number of sessions, treatment duration (weeks), length of each session, type of intervention, target population, and age (see **Table 3**). Regarding the number of sessions, <8 sessions (SMD = -1.09, 95% CI -2.44~0.27, P = 0.12) and 8–12 sessions (SMD = -0.53, 95% CI -1.16~0.11, P = 0.10) did not reach statistical significance, whereas >12 sessions showed a statistically significant effect(SMD = -0.53, 95% CI -0.87~-0.20, P = 0.002).

**Table 3:** Subgroup analyses of anxiety outcomes associated with visual art therapy in adults.

Subgroups	No. of studies	Total sample (I+C)	Heterogeneity		SMD (95% CI)	Meta-analysis results	
			I^2^ (%)	P-value		Z	P
Treatment Frequency (sessions)							
<8	2	95	89	0.003	-1.09 [-2.44, 0.27]	1.57	0.12
8-12	2	127	66	0.08	-0.53 [-1.16, 0.11]	1.63	0.10
>12	2	144	0	0.42	-0.53 [-0.87, -0.20]	3.14	0.002
Treatment duration (weeks)							
≤6	4	263	74	0.009	-0.90 [-1.42, -0.38]	3.40	0.0007
>6	2	103	0	0.64	-0.28 [-0.67, 0.11]	1.40	0.16
Session Duration (min)							
≤60	3	175	80	0.007	-1.00 [-1.74, -0.26]	2.65	0.008
>60	2	103	0	0.64	-0.28 [-0.67, 0.11]	1.40	0.16
Type of Intervention							
PT	4	263	74	0.009	-0.90 [-1.42, -0.38]	3.40	0.0007
MVAT	2	103	0	0.64	-0.28 [-0.67, 0.11]	1.40	0.16
Population							
D	3	171	3	0.36	-0.58 [-0.89, -0.27]	3.68	0.0002
OCP	3	195	87	0.0006	-0.85 [-1.69, -0.02]	2.00	0.05
Ages							
<65	4	263	74	0.009	-0.90 [-1.42, -0.38]	3.40	0.0007
≥65	2	103	0	0.64	-0.28 [-0.67, 0.11]	1.40	0.16

I+C, intervention group plus control group; SMD, standardized mean difference; CI, confidence interval; PT, painting therapy; MVAT, multi-media visual art therapy; D, adults with depression; OCP, other clinical populations.

In terms of treatment duration, ≤6 weeks(SMD = -0.90, 95% CI -1.42~-0.38, P = 0.0007) showed significant improvement, whereas >6 weeks did not reach statistical significance (SMD = -0.28, 95% CI -0.67~0.11, P = 0.16). With respect to the length of each session, ≤60 minutes(SMD = -1.00, 95% CI -1.74~-0.26, P = 0.008)showed a statistically significant effect, whereas >60 minutes did not (SMD = -0.28, 95% CI -0.67~0.11, P = 0.16).

When stratified by type of intervention, drawing-based therapy showed a statistically significant effect (SMD = -0.90, 95% CI: -1.42~-0.38, P = 0.0007), whereas multimodal visual art therapy did not (SMD = -0.28, 95% CI: -0.67~0.11, P = 0.16). When stratified by target population, the subgroup of individuals with depression showed significant improvement (SMD = -0.58, 95% CI: -0.89 to -0.27, P = 0.0002); for other clinical populations, the effect size was SMD = -0.85 (95% CI: -1.69 to -0.02, P = 0.05), indicating a borderline level of significance.

Age-stratified analyses showed a statistically significant effect in participants aged <65 years (SMD = -0.90, 95% CI: -1.42~-0.38, P = 0.0007), whereas no significant effect was observed in those aged ≥65 years (SMD = -0.28, 95% CI: -0.67~0.11, P = 0.16).

## 4 Discussion

Based on 12 randomized controlled trials involving a total of 741 adult participants, this study systematically evaluated the intervention effects of VAT on depressive and anxiety symptoms. The meta-analysis showed that VAT significantly improved depressive and anxiety symptoms in adults, indicating that, as a non-pharmacological psychological intervention, VAT plays a positive role in emotion regulation.

### 4.1 Effects of visual art therapy on depressive symptoms in adults and related influencing factors

The therapeutic mode of VAT differs from traditional verbal psychotherapy in that its mechanisms of action may involve activating neural pathways related to perception, sensorimotor integration, and visuospatial processing, thereby providing a nonverbal channel for the externalization of emotional experiences that are difficult to articulate (32, 33). Preliminary evidence from neuroimaging studies suggests that engagement in visual art-making may modulate hyperactivation of the amygdala, enhance top–down control of the limbic system by the prefrontal cortex, and improve functional connectivity within the default mode network (DMN) (34–36). These neural circuits constitute the core pathophysiological substrates of emotional dysregulation and ruminative thinking in patients with depression (37, 38). At the same time, it has been hypothesized that the flow experience during art-making is accompanied by activation of reward pathways (e.g., ventral striatum and anterior cingulate cortex), which can induce dopamine release and may directly counteract anhedonia, a core symptom of depression (39–41). It should be noted that the above neurobiological explanations are primarily based on basic research in related fields and theoretical inferences, rather than direct evidence from the clinical trials included in this study. The core findings of the present study are still mainly grounded in the effect sizes for improvements in clinical symptoms.

The results of this meta-analysis showed that VAT had a significant effect on depressive symptoms in adults (SMD = -0.81, 95% CI: -1.16~-0.46, Z = 4.52, P < 0.00001), which was largely consistent with the overall effect size (SMD = -0.73) reported by Han et al. (2024) (21).In this study, subgroup analyses were further conducted to refine the effects according to treatment duration (weeks), number of sessions, length of each session, type of intervention, population characteristics, and age.

The subgroup analyses indicated that interventions lasting ≤6 weeks and 7–12 weeks both significantly improved depressive symptoms, with 7–12 weeks showing a superior effect (SMD = -2.00); in contrast, interventions longer than 12 weeks had a smaller effect size and were at a borderline level of significance (SMD = -0.39, P = 0.05). This finding supports the view from previous systematic reviews that intervention durations of no more than 12 weeks yield better outcomes (21, 42). However, as this subgroup included only three studies and exhibited substantial heterogeneity (I² = 92%), the corresponding results should be interpreted with caution. Subgroup analyses of the number of sessions showed that interventions with ≤12 sessions had significant effects (<8 sessions: SMD = -0.79; 8–12 sessions: SMD = -0.81), whereas >12 sessions did not reach statistical significance (SMD = -0.90, P = 0.08). Based on these results, it can be inferred that improvement in depressive symptoms requires interventions of sufficient duration to establish new cognitive and behavioral patterns (43). Short-term interventions may be insufficient to stabilize functional connectivity between the prefrontal cortex and limbic system and thus fail to produce cumulative neuroplastic changes (44). However, beyond a certain number of sessions, diminishing marginal returns or even adverse effects may occur, potentially due to treatment fatigue, reduced motivation, or a discrepancy between participants’ expectations and actual improvement (45).

For the length of each session, both ≤60 minutes (SMD = -0.98) and >60 minutes (SMD = -0.82) showed significant effects, with the former having a larger absolute effect size. A single-session duration within 60 minutes may help participants maintain a higher level of attention and engagement in the creative process, whereas sessions longer than 60 minutes may weaken the intervention effects due to fatigue, physical discomfort (particularly in older adults or those with comorbid physical illnesses), and increased cognitive load (46, 47).

Among the intervention modalities, multimodal visual art therapy showed the most pronounced effect size (SMD = -0.98), followed by free drawing therapy (SMD = -0.84), whereas structured pattern drawing therapy had a relatively smaller effect size (SMD = -0.48), and clay art therapy did not reach statistical significance. This gradient may be related to the depth of cognitive processing and the degree of emotional expressiveness elicited by different art forms (48). Multimodal visual art therapy allow participants to switch freely among different media such as drawing, collage, and photography, accommodating individual differences in expressive preferences and thereby enhancing the accessibility and personalization of treatment (49, 50). Free drawing therapy, implemented without fixed templates, grants participants a high degree of freedom in choosing themes and media, which facilitates spontaneous emotional externalization and the construction of personal meaning (51). Structured pattern drawing (e.g., mandala coloring) helps individuals establish an inner sense of safety through its inherent order and protective boundaries, regulating depressive mood with a relatively low cognitive load (52). Clay art therapy involves the integration of tactile, proprioceptive, and fine motor experiences; its three-dimensional modeling process is conducive to embodied emotion regulation and the expression of trauma-related metaphors (53). The lack of statistical significance in this subgroup may be primarily attributable to the limited number of included studies (only two) and the small overall sample size.Given the exploratory nature of the subgroup analyses, the limited number of studies in some subgroups, and the presence of heterogeneity, these findings should be interpreted with caution.

In terms of population characteristics, both clinical populations with depression (SMD = -0.57) and community-dwelling adults (SMD = -1.84) showed significant improvement, with a larger effect size observed in community samples. This difference may reflect the influence of baseline depression severity on effect sizes: community samples predominantly present with subclinical depressive symptoms and may be more sensitive to low-intensity interventions, whereas patients with clinical depression have more severe symptoms and may require more intensive or longer-term interventions to achieve comparable improvements (54). The subgroup of post-stroke survivors did not show a statistically significant difference (SMD = -1.13, P = 0.21), which may be related to limitations in VAT engagement caused by neurological deficits, aphasia, or cognitive impairment in this population (55, 56). In light of the limited number of studies in this subgroup, these findings are exploratory and should be interpreted with caution; potential differences in treatment effects across populations require further empirical verification.

Age-stratified analyses showed statistically significant effects in both participants aged <65 years (SMD = -0.81) and those aged ≥65 years (SMD = -0.83), suggesting that VAT is applicable across different adult age groups. In contrast, the study by Han et al. (2024) (21) did not find a statistically significant effect in participants aged ≥65 years. These discrepancies may be related to differences in eligibility criteria and the inclusion of newly published evidence. Given the exploratory nature of the subgroup analyses, these findings should be interpreted with caution.

### 4.2 Effects of visual art therapy on anxiety symptoms in adults and related influencing factors

Anxiety is characterized by excessive worry about potential threats, a state that continuously occupies attentional resources and leads to reduced cognitive control (57). Visual art-making requires intensive sensorimotor integration and sustained attentional focus, thereby reallocating attentional resources away from anxiety-provoking stimuli toward the creative task itself (58). Tactile feedback and the execution of fine motor movements can effectively reduce cortisol levels and enhance parasympathetic activity, thereby alleviating anxiety at the physiological level (59, 60). Previous studies have indicated that manual art-based activities can significantly reduce state anxiety by activating sensorimotor integration circuits (61).

The results of this meta-analysis showed that VAT significantly improved anxiety symptoms in adults (SMD = -0.69, 95% CI: -0.90 to -0.48, Z = 6.31, P < 0.00001) (62), with a smaller effect size than that reported by Huang et al. (2025) (SMD = -1.31). In Huang et al.’s review, a relatively high proportion of studies used waitlist controls, whereas in the present study anxiety was treated as a secondary outcome, the proportion of participants with anxiety was lower, and the controls received usual care, leading to a relatively conservative effect estimate (63).

Analyses of the number of sessions showed that >12 sessions were significantly effective in improving anxiety symptoms (SMD = -0.53, P = 0.002), whereas interventions with ≤12 sessions did not reach statistical significance (<8 sessions: SMD = -1.09, P = 0.12; 8–12 sessions: SMD = -0.53, P = 0.10). Improvement in anxiety symptoms may require higher-frequency treatment, which is consistent with the persistent and recurrent nature of anxiety (64). This finding echoes the widely observed dose–response relationship in the field of psychological interventions, whereby the number of sessions is a key factor modulating treatment efficacy (65, 66). However, this finding is based on a small number of included studies, and the subgroup analyses are exploratory in nature; therefore, the corresponding conclusions should be interpreted with caution.

Analyses of treatment duration and length of each session showed that interventions lasting ≤6 weeks were significantly effective in improving anxiety symptoms (SMD = -0.90), whereas those >6 weeks did not reach statistical significance; similarly, sessions of ≤60 minutes were significantly effective (SMD = -1.00), whereas those >60 minutes were not. The results for these two dose parameters were consistent: effects were already achieved at lower doses (≤6 weeks, ≤60 minutes), and increasing the dose did not confer additional benefits and even tended toward non-significance at higher doses. Individuals with anxiety often exhibit heightened physiological arousal and hypervigilance to internal sensations; excessively long single sessions are likely to induce fatigue and anticipatory anxiety, thereby attenuating the therapeutic effect (67). The study by Kaimal et al. (59) (2016) further demonstrated that a single 45-minute session of visual art-making significantly reduced cortisol levels, providing neuroendocrinological evidence at the physiological level for the effectiveness of short-term interventions (≤60 minutes). These findings numerically favor intervention patterns characterized by a “shorter duration” and “shorter single-session length”; however, given the limited number of available studies, the corresponding conclusions should be interpreted with caution.

Among the intervention modalities, drawing-based therapy significantly improved anxiety (SMD = -0.90), whereas the multimodal visual art therapy subgroup did not reach statistical significance. This discrepancy may be attributable to the small number of studies in the multimodal visual art therapy subgroup (only two) and substantial heterogeneity in intervention protocols. Drawing-based interventions typically have clearly defined task boundaries and well-specified procedures (68), which help reduce decision-making load and facilitate entry into a flow state, thereby enabling individuals to temporarily disengage from anxiety-provoking stimuli (69).

In terms of population characteristics, the effect of VAT on anxiety symptoms was stable in individuals with depression (SMD = -0.58). The subgroup of other clinical populations (post-stroke survivors, individuals with generalized anxiety disorder, and community correction participants) showed a relatively large effect size, but at a borderline level of significance (SMD = -0.85, P = 0.05), which may reflect differential responses to the intervention due to heterogeneity in diagnostic categories and anxiety severity (70). Previous studies have suggested that conditions such as generalized anxiety disorder may require higher treatment doses to achieve stable effects (22). Given the limited number of studies included in the subgroups and the heterogeneous composition of the populations, these findings are exploratory and should be interpreted with caution.

Age-stratified analyses showed that anxiety symptoms improved significantly in adults aged <65 years (SMD = -0.90), whereas no statistically significant effect was observed in older adults aged ≥65 years. The clear benefits observed in younger adults may be related to their higher acceptance of nonverbal interventions and greater emotional awareness, whereas the lack of significant effects in older adults may be influenced by a stronger tendency toward somatic manifestations of anxiety and limitations in intervention engagement due to comorbid cognitive impairment (23, 71). Given the limited number of studies included in the subgroups, these findings are exploratory and should be interpreted with caution.

## 5 Conclusions

This study systematically synthesized evidence from 12 randomized controlled trials involving a total of 741 adult participants and found that visual art therapy exerted beneficial effects in alleviating depressive symptoms (SMD = -0.81) and anxiety symptoms (SMD = -0.69) in adults. Subgroup analyses suggested that, for depressive symptoms, relatively larger effect sizes were observed when the intervention duration was <12 weeks, the total number of sessions was kept within 12, and the single-session length did not exceed 60 minutes. For anxiety symptoms, larger effect sizes were associated with intervention protocols characterized by higher frequency (>12 sessions), shorter overall duration (≤6 weeks), and shorter single-session length (≤60 minutes). As a non-pharmacological intervention, VAT may have potential as an adjunctive approach for alleviating depressive and anxiety symptoms in adults. However, in view of the considerable heterogeneity and methodological limitations of the included studies, further high-quality randomized controlled trials are needed to confirm these findings.

This study has several limitations: (1) Owing to the inherent characteristics of visual art therapy, none of the included studies could implement blinding of participants or interventionists, and allocation concealment was not reported, which may have introduced performance bias and selection bias; (2) there were considerable differences across studies in intervention protocols, types of control conditions, and outcome measures, resulting in substantial heterogeneity in the pooled effects; (3) because only a small number of studies were included in each subgroup and the study designs varied, the subgroup findings are exploratory and require further verification. For example, the included populations encompassed diverse groups such as individuals with depressive disorders, post-stroke survivors, people with mild cognitive impairment, and community samples, which differ markedly in symptom profiles, baseline severity, treatment needs, and potential responsiveness to intervention, thereby limiting the stability and generalizability of the results; (4) the specific procedural details of visual art therapy lack standardized guidelines, and none of the included studies systematically reported adverse events, withdrawals due to adverse events, or safety monitoring procedures, which weakens the assessment of safety; (5) the distribution of active versus passive control conditions was unbalanced across the included trials, making it difficult to support subgroup analyses and leaving the moderating effect of control type on effect sizes unclear. Future research should therefore involve larger, rigorously designed randomized controlled trials and adopt standardized reporting of intervention parameters, in order to further clarify the optimal dose–response relationship of VAT and its applicability across different populations.

## Data Availability

The original contributions presented in the study are included in the article/**Supplementary Material**. Further inquiries can be directed to the corresponding author.
