# Supplementary material for: Effects of visual art therapy on depressive symptoms in adults: a systematic review and meta-analysis
Source: Front Psychiatry. 2026 Jul 22;17:1877502. doi: 10.3389/fpsyt.2026.1877502 (PMC13437678; doi:10.3389/fpsyt.2026.1877502)
Supplement: Supplementary file 1 [file Table1.docx]

| **Table S1 The search strategy** | | |
| --- | --- | --- |
| **Datebases** | **Searching strategy** | **Literature number** |
| **Pubmed** | ((((((((((((((((((((((((Art Therapy[MeSH Terms]) OR (Art Therapy[Title/Abstract])) OR (Therapy, Art[Title/Abstract])) OR (Art Therapies[Title/Abstract])) OR (Therapies, Art[Title/Abstract])) OR (Visual Arts Therapy[Title/Abstract])) OR (Visual Art[Title/Abstract])) OR (painting[Title/Abstract])) OR (Collage making[Title/Abstract])) OR (creative arts[Title/Abstract])) OR (Sketching[Title/Abstract])) OR (pottery[Title/Abstract])) OR (ceramics[Title/Abstract]))) OR (sculpture[Title/Abstract])) OR (Craft[Title/Abstract])) OR (Colouring[Title/Abstract])) OR (Sandpainting[Title/Abstract])) OR (Mandala Painting[Title/Abstract])) OR (Art[MeSH Terms])) OR (Art[Title/Abstract])) OR (Arts[Title/Abstract])) AND (((Adult[MeSH Terms]) OR (Adult[Title/Abstract])) OR (Adults[Title/Abstract]))) AND (((((((((((((((((((((((((((((Depression[MeSH Terms]) OR (Depression[Title/Abstract])) OR (Depressive Symptoms[Title/Abstract])) OR (Depressive Symptom[Title/Abstract])) OR (Symptom, Depressive[Title/Abstract])) OR (Emotional Depression[Title/Abstract])) OR (Depression, Emotional[Title/Abstract])) OR (Depressive Disorder[MeSH Terms])) OR (Depressive Disorder[Title/Abstract])) OR (Depressive Disorders[Title/Abstract])) OR (Disorder, Depressive[Title/Abstract])) OR (Disorders, Depressive[Title/Abstract])) OR (Depression Disorder[Title/Abstract])) OR (Disorders, Depression[Title/Abstract])) OR (Neurosis, Depressive[Title/Abstract])) OR (Depressive Neuroses[Title/Abstract])) OR (Neuroses, Depressive[Title/Abstract])) OR (Depression, Endogenous[Title/Abstract])) OR (Depressions, Endogenous[Title/Abstract])) OR (Endogenous Depression[Title/Abstract])) OR (Melancholia[Title/Abstract])) OR (Unipolar Depression[Title/Abstract])) OR (Depression, Unipolar[Title/Abstract])) OR (Depressive Syndrome[Title/Abstract])) OR (Syndrome, Depressive[Title/Abstract])) OR (Syndromes, Depressive[Title/Abstract])) OR (Depression, Neurotic[Title/Abstract])) OR (Depressions, Neurotic[Title/Abstract])) OR (Neurotic Depression[Title/Abstract]))) AND (((Randomised controlled trial[MeSH Terms]) OR (Randomised controlled trial[Title/Abstract])) OR (RCT[Title/Abstract])) | **64** |
| **Web of science** | #1 TS=("art therapy" OR "therapy art" OR "art therapies" OR "therapies art" OR (visual* NEAR/3 "arts therapy")OR "visual art" OR painting OR "collage making" OR "creative arts" OR sketching OR pottery OR ceramics OR sculpture OR craft* OR colouring OR coloring OR "mandala painting" OR art OR arts) 2614746  #2 TS=( adult OR adults) 2439762  #3 TS=(depress* OR "depressive symptom" OR "depressive symptoms" OR "depressive disorder" OR "depressive disorders" OR "depressive neurosis" OR "depressive neuroses" OR "neurotic depression" OR "endogenous depression" OR "endogenous depressions" OR melanchol* OR "unipolar depression" OR "depressive syndrome" OR "depressive syndromes") 1009701  #4 TS=("randomized controlled trial" OR "randomised controlled trial" OR "randomized clinical trial" OR "randomised clinical trial" OR "randomized controlled trials" OR "randomised controlled trials" OR (random* NEAR/3 (trial* OR clinical)) OR RCT OR RCTs) 852430  #4 AND #3 AND #2 AND #1 230 | **230** |
| Embase |  | **630** |
| Cochrane Library | ID Search Hits  #1 (Art Therapy):ti,ab,kw OR (Therapy, Art):ti,ab,kw OR (Art Therapies):ti,ab,kw OR (Therapies, Art):ti,ab,kw OR (Visual Arts Therapy):ti,ab,kw (Word variations have been searched) 7974  #2 (Visual Art):ti,ab,kw OR (painting):ti,ab,kw OR (Collage making):ti,ab,kw OR (creative arts):ti,ab,kw OR (Sketching):ti,ab,kw (Word variations have been searched) 1953  #3 (pottery):ti,ab,kw OR (ceramics):ti,ab,kw OR (Colouring):ti,ab,kw OR (sculpture):ti,ab,kw OR (Craft):ti,ab,kw (Word variations have been searched) 22271  #4 (Mandala Painting):ti,ab,kw OR (Sandpainting):ti,ab,kw OR (Mandala Painting):ti,ab,kw (Word variations have been searched) 49  #5 (Arts):ti,ab,kw OR (Art):ti,ab,kw (Word variations have been searched) 12886  #6 (Adult):ti,ab,kw OR (Adults):ti,ab,kw (Word variations have been searched) 945596  #7 #1 OR #2 OR #3 OR #4 OR #5 35634  #8 (Depression):ti,ab,kw OR (Depressive Symptoms):ti,ab,kw OR (Depressive Symptom):ti,ab,kw OR (Symptom, Depressive):ti,ab,kw OR (Emotional Depression):ti,ab,kw (Word variations have been searched) 131706  #9 (Depression, Emotional):ti,ab,kw OR (Depressive Disorder):ti,ab,kw OR (Depressive Disorders):ti,ab,kw OR (Disorder, Depressive):ti,ab,kw OR (Disorders, Depressive):ti,ab,kw (Word variations have been searched) 64517  #10 (Depression Disorder):ti,ab,kw OR (Disorders, Depression):ti,ab,kw OR (Neurosis, Depressive):ti,ab,kw OR (Depressive Neuroses):ti,ab,kw OR (Neuroses, Depressive):ti,ab,kw (Word variations have been searched) 57213  #11 (Depression, Endogenous):ti,ab,kw OR (Depressions, Endogenous):ti,ab,kw OR (Endogenous Depression):ti,ab,kw OR (Melancholia):ti,ab,kw OR (Unipolar Depression):ti,ab,kw (Word variations have been searched) 3574  #12 (Depression, Unipolar):ti,ab,kw OR (Depressive Syndrome):ti,ab,kw OR (Syndrome, Depressive):ti,ab,kw OR (Syndromes, Depressive):ti,ab,kw OR (Depression, Neurotic):ti,ab,kw (Word variations have been searched) 13603  #13 (Depressions, Neurotic):ti,ab,kw OR (Neurotic Depression):ti,ab,kw (Word variations have been searched) 325  #14 #8 OR #9 OR #10 OR #11 OR #12 OR #13 131736  #15 #7 AND #6 AND #14 990  #16 (Randomised controlled trial):ti,ab,kw OR (RCT):ti,ab,kw (Word variations have been searched) 948390  #17 #15 AND #16 636 | **631** |
| CNKI | (TKA%=艺术疗愈 OR TKA%=艺术治疗 OR TKA%=视觉艺术治疗 OR TKA%=视觉艺术疗法  OR TKA%=曼陀罗绘画治疗 OR TKA%=曼陀罗疗法  OR TKA%=绘画治疗 OR TKA%=美术治疗  OR TKA%=拼贴 OR TKA%=手工治疗 OR TKA%=雕塑 OR TKA%=陶艺)  AND  (TKA%=老年 OR TKA%=老年人)  AND  (TKA%=情绪性抑郁 OR TKA%=情绪性抑郁症 OR TKA%=情绪抑郁 OR TKA%=抑郁症状  OR TKA%=抑郁障碍 OR TKA%=抑郁病人  OR TKA%=抑郁) | **242** |
| Wanfang Data | ("艺术疗愈" OR "艺术治疗" OR "视觉艺术治疗" OR "视觉艺术疗法" OR "视觉艺术"  OR "曼陀罗绘画治疗" OR "曼陀罗疗法" OR "曼陀罗画" OR "绘画治疗" OR "美术治疗"  OR "手工治疗" OR "拼贴" OR "雕塑" OR "沙画" OR "陶艺" OR "素描" OR "创意艺术")  AND  ("成年人" OR "成人")  AND  ("抑郁症" OR "抑郁" OR "抑郁情绪" OR "情绪性抑郁" OR "情绪性抑郁症" OR "情绪抑郁" OR "抑郁症状" OR "抑郁障碍" OR "抑郁病人" OR "抑郁") | **2** |
| CBM | (TM=("艺术疗愈" OR "艺术治疗" OR "视觉艺术治疗" OR "视觉艺术疗法" OR "视觉艺术" OR "曼陀罗绘画治疗" OR "曼陀罗疗法" OR "曼陀罗画" OR "绘画治疗" OR "美术治疗" OR "手工治疗" OR "拼贴" OR "雕塑" OR "陶艺") AND (TM=("成年人")) AND (TM=("情绪性抑郁" OR "情绪性抑郁症" OR "情绪抑郁" OR "抑郁症状" OR "抑郁障碍" OR "抑郁病人" OR "抑郁")) | **2** |
| VIP Database | ((T="艺术疗愈" OR K="艺术疗愈" OR R="艺术疗愈")  OR (T="艺术治疗" OR K="艺术治疗" OR R="艺术治疗")  OR (T="视觉艺术治疗" OR K="视觉艺术治疗" OR R="视觉艺术治疗")  OR (T="视觉艺术疗法" OR K="视觉艺术疗法" OR R="视觉艺术疗法")  OR (T="视觉艺术" OR K="视觉艺术" OR R="视觉艺术")  OR (T="曼陀罗绘画治疗" OR K="曼陀罗绘画治疗" OR R="曼陀罗绘画治疗")  OR (T="曼陀罗疗法" OR K="曼陀罗疗法" OR R="曼陀罗疗法")  OR (T="曼陀罗画" OR K="曼陀罗画" OR R="曼陀罗画")  OR (T="绘画治疗" OR K="绘画治疗" OR R="绘画治疗")  OR (T="美术治疗" OR K="美术治疗" OR R="美术治疗")  OR (T="手工治疗" OR K="手工治疗" OR R="手工治疗")  OR (T="拼贴" OR K="拼贴" OR R="拼贴")  OR (T="雕塑" OR K="雕塑" OR R="雕塑")  OR (T="陶艺" OR K="陶艺" OR R="陶艺"))  AND  ((T="成年人" OR K="成年人" OR R="成年人"))  AND  ((T="情绪性抑郁" OR K="情绪性抑郁" OR R="情绪性抑郁")  OR (T="情绪性抑郁症" OR K="情绪性抑郁症" OR R="情绪性抑郁症")  OR (T="情绪抑郁" OR K="情绪抑郁" OR R="情绪抑郁")  OR (T="抑郁症状" OR K="抑郁症状" OR R="抑郁症状")  OR (T="抑郁障碍" OR K="抑郁障碍" OR R="抑郁障碍")  OR (T="抑郁病人" OR K="抑郁病人" OR R="抑郁病人")  OR (T="抑郁" OR K="抑郁" OR R="抑郁")) | **0** |
