# Supplementary material for: Effects of visual art therapy on depressive symptoms in adults: a systematic review and meta-analysis
Source: Front Psychiatry. 2026 Jul 22;17:1877502. doi: 10.3389/fpsyt.2026.1877502 (PMC13437678; doi:10.3389/fpsyt.2026.1877502)
Supplement: Supplementary file 2 [file Table2.docx]

**Supplementary Table S2**

**Table S2 GRADE Evidence Quality Assessment**

| **Outcome** **indicator** | **Participants** **(studies)** | **Risk of** **bias** | **Inconsistency** | **Indirectness** | **Imprecision** | **Publication** **bias** | **SMD (95% CI)** | **Certainty** |
| --- | --- | --- | --- | --- | --- | --- | --- | --- |
| Depression | 741 (12 RCTs) | serious^a^ | not serious | not serious | not serious | none | -0.81 [-1.16, -0.46] | ⊕⊕⊕○ Moderate^a^ |
| Anxiety | 366 (6 RCTs) | serious^a^ | serious^b^ | not serious | not serious | none | -0.69 [-0.90, -0.48] | ⊕⊕○○ Low^a,b^ |

^a^ Downgraded for study limitations: included trials were limited by insufficient reporting of allocation concealment and blinding, and all trials were unable to blind participants and intervention providers due to the nature of visual art therapy.

^b^ Downgraded for inconsistency due to substantial heterogeneity across studies.

**Supplementary Table .** Summary of findings and certainty of evidence using GRADE
